# Achieving Lower Nitrogen Balance and Higher Nitrogen Recovery Efficiency Reduces Nitrous Oxide Emissions in North America's Maize Cropping Systems

**DOI:** 10.3389/fpls.2017.01080

**Published:** 2017-06-23

**Authors:** Rex A. Omonode, Ardell D. Halvorson, Bernard Gagnon, Tony J. Vyn

**Affiliations:** ^1^Department of Agronomy, Purdue UniversityWest Lafayette, IN, United States; ^2^United States Department of Agriculture - Agricultural Research Service (USDA-ARS)Fort Collins, CO, United States; ^3^Soils and Crops Research and Development Centre, Agriculture and Agri-Food CanadaQuebec City, QC, Canada

**Keywords:** aboveground N uptake, grain N uptake, net nitrogen balance, nitrogen recovery efficiency, nitrous oxide emission, surplus nitrogen

## Abstract

Few studies have assessed the common, yet unproven, hypothesis that an increase of plant nitrogen (N) uptake and/or recovery efficiency (NRE) will reduce nitrous oxide (N_2_O) emission during crop production. Understanding the relationships between N_2_O emissions and crop N uptake and use efficiency parameters can help inform crop N management recommendations for both efficiency and environmental goals. Analyses were conducted to determine which of several commonly used crop N uptake-derived parameters related most strongly to growing season N_2_O emissions under varying N management practices in North American maize systems. Nitrogen uptake-derived variables included total aboveground N uptake (TNU), grain N uptake (GNU), N recovery efficiency (NRE), net N balance (NNB) in relation to GNU [NNB_(GNU)_] and TNU [NNB_(TNU)_], and surplus N (SN). The relationship between N_2_O and N application rate was sigmoidal with relatively small emissions for N rates <130 kg ha^−1^, and a sharp increase for N rates from 130 to 220 kg ha^−1^; on average, N_2_O increased linearly by about 5 g N per kg of N applied for rates up to 220 kg ha^−1^. Fairly strong and significant negative relationships existed between N_2_O and NRE when management focused on N application rate (*r*^2^ = 0.52) or rate and timing combinations (*r*^2^ = 0.65). For every percentage point increase, N_2_O decreased by 13 g N ha^−1^ in response to N rates, and by 20 g N ha^−1^ for NRE changes in response to rate-by-timing treatments. However, more consistent positive relationships (*R*^2^ = 0.73–0.77) existed between N_2_O and NNB_(TNU)_, NNB_(GNU)_, and SN, regardless of rate and timing of N application; on average N_2_O emission increased by about 5, 7, and 8 g N, respectively, per kg increase of NNB_(GNU)_, NNB_(TNU)_, and SN. Neither N source nor placement influenced the relationship between N_2_O and NRE. Overall, our analysis indicated that a careful selection of appropriate N rate applied at the right time can both increase NRE and reduce N_2_O. However, N_2_O reduction benefits of optimum N rate-by-timing practices were achieved most consistently with management systems that reduced NNB through an increase of grain N removal or total plant N uptake relative to the total fertilizer N applied to maize. Future research assessing crop or N management effects on N_2_O should include N uptake parameter measurements to better understand N_2_O emission relationships to plant NRE and N uptake.

## Introduction

North America (Canada, Mexico, and the United States) plays an important role in the world's maize production and the consequent nitrous oxide emissions arising from high nitrogen (N) fertilizer applied during maize production. In 2014–2015, North America (United States and Canada) accounted for about 37% of the World's 1015.6 million metric tons of maize produced on 34.9 million hectares (World = 179.8 million hectares; USDA/FAS, 2017) and consumed about 13% (14.1 million) of the 113 million metric tons of fertilizer N consumed worldwide (FAO, 2016). In the United States, about 40% or 5.6 million of the total 12.8 million metric tons of the N fertilizers consumed annually in 2012–2014 was applied to maize. Maize cropping systems in North America are thus of major concern with respect to nitrous oxide (N_2_O) emissions.

Nitrous oxide is both an important ozone-depleting chemical (Ravishankara et al., 2009), and a major greenhouse gas that is believed to contribute to global climate change with a potency that is about 310 times the global warming potential of CO_2_ (IPCC, 2007). In agricultural soils, N_2_O is produced predominantly through bacterial-mediated transformations of inorganic nitrogen (N), but the quantity and intensity of N_2_O emission so emitted is dependent on soil and N fertilizer management options applied, and their interactions with environmental factors during crop production (Venterea et al., 2012).

Several reviews and/or meta-analyses have synthesized the body of research and identified specific N management practices including rate, type/source and placement, and tillage systems that have the potential to reduce N_2_O in the context of broader agro-ecological systems (Eichner, 1990; Akiyama et al., 2010; Decock, 2014; Snyder et al., 2014). In recent times, crop yield and/or yield-related N_2_O parameters have been included in field experiments and research reviews to relate emissions with the agronomic parameters (Mosier et al., 2006; Van Groenigen et al., 2010; Abalos et al., 2016) in attempts to identify a suitable practice or combination of practices that reduced N_2_O loss without adverse effects on yield. For example, one report examined how the combination of reduced N rate, nitrification inhibitor, and N timing potentially reduced N_2_O loss without a reduction in grain yield (Abalos et al., 2016). However, these reviews also highlighted a lack of studies that also included the critical information of treatment effects on crop N uptake and use efficiency (Decock, 2014).

Nitrogen management changes in rate, source, timing, and placement (applied as single factors, or in some combination) are often recommended because they are believed to have the potential to reduce N_2_O emissions and maintain yields through improved total aboveground N uptake (TNU) and/or N use efficiency (NRE: TNU in fertilized plot *minus* TNU in control plots relative to fertilizer N applied), or a decrease of surplus N (SN: fertilizer N applied *minus* TNU; Snyder et al., 2014). Therefore, although unproven, the common hypothesis is that increased TNU or NRE, or a decrease of SN, will be associated with reduced N_2_O emissions due to a decrease in available soil inorganic N, from which much of N_2_O derives via soil nitrification and denitrification processes. Yet, very few studies have attempted to link N_2_O emissions to TNU, NRE, and SN (Mosier et al., 2006; Van Groenigen et al., 2010; Venterea et al., 2016), perhaps because the relevant data to relate these parameters are seldom collected and/or reported for the same experiment.

While NRE and SN are good parameters to evaluate a cropping system's effects on N_2_O, Grassini and Cassman (2012) suggested that use of a (net) N balance (NNB) approach (NNB: fertilizer N + recoverable manure N + legume N fixation – N removed by crops) for estimating soil N_2_O emissions was probably preferable to the SN or the IPCC's emission factor (EF) method. This was because EF varies significantly with N application, and N_2_O losses are related to the amount of excess N in the system rather than to in-season N inputs from fertilizer application *per se* (Cavigelli et al., 2012). The latter is especially applicable to most of North America's maize systems when maize is grown in rotation with legumes like soybean or alfalfa and where manure may also be applied to supplement N fertilizer application. These practices represent significant sources of N input into production systems and can affect the balance of N available for bacterial denitrification and N uptake, and subsequently the quantity of N_2_O emitted into the atmosphere. To the best of our knowledge, little or no studies have been conducted that related N_2_O to the production system's N balance; therefore, the nature and extent of such relationships remain largely unknown.

The main objectives of this study were to assess relationships between growing season N_2_O and crop N uptake-related metrics (TNU/GNU, NRE, NNB, and SN) and determine which of these parameters related most strongly and consistently to N_2_O emissions under commonly applied N management practices (rate, source, timing, placement) in North America's maize systems, using data synthesized from field experiments where N_2_O emission and N uptake were measured in the same site-years. We hypothesize that including these variables in models to evaluate a cropping system's effects on N_2_O provide a more holistic approach to improving our understanding of these relationships, and helps to better guide selection of N management options for this important maize production region. We also examined synchronies between N application rate, yield, and NRE as a pathway to better understand N_2_O versus N uptake dynamics, and to provide needed guidance to crafting policies and management practices with the potential to maintain yield and reduce N_2_O emissions during maize production.

## Materials and methods

### Data collection, processing, and structure

The data used for this analysis were obtained from researchers across North America following a preliminary literature survey of peer-reviewed publications that reported N_2_O emissions for North America's maize production systems to identify experiments where N uptake was possibly measured along with the reported N_2_O emission in the same study. Following this survey, we requested and received from authors plot or replicate-level maize grain yield, total above ground whole-plant N uptake (TNU), and/or grain N uptake (GNU) data that were measured along with the original N_2_O data. Altogether, a total of 1,375 plot-level observations (432 mean observations, averaged over replicates) of cumulative seasonal N_2_O emission and their corresponding grain yield, GNU, and/or TNU data points derived from various N management systems across North America were received. A close observation of the data showed that 90% of the N_2_O data received have been published in 23 peer-reviewed publications (10% unpublished, derived from 2 studies in Indiana). Similarly, 63% of the N uptake data were published either along with their corresponding N_2_O data or separately in different journals, while 37% were unpublished. Details on data sources, locations and year of experiments, and N management practices are shown in Supplemental Table S1.

To be included in the final dataset, both N_2_O and maize N uptake data must have originated from the same experiment conducted with ≥3 replicates for ≥2 years, and where N_2_O emission was measured at least weekly for the greater part of the growing season using standard methods (vented chamber or micrometeorology procedures). However, in one instance N uptake data from one growing season was included in the dataset because the experiment involved multiple N rates and application timings (Venterea and Coulter, 2015). The data was further processed and observations from experiments that did not include control plots i.e., where no N was applied (e.g., Adviento-Borbe et al., 2007) were removed. This was because certain parameters such as fertilizer induced N_2_O emission (FIE) and NRE could not be calculated for those experiments or locations. Data from experiments that involved manure applied at a N single rate (4 mean observations; Sistani et al., 2011; Halvorson et al., 2016a,b) were also excluded because they were too small a sample from which to infer N_2_O consequences from an N source distinct management option. Similarly, data from Quebec City, Quebec (Gagnon et al., 2011) were removed because the N_2_O values from this location were several orders of magnitude greater (mean = 17.7 kg ha^−1^; range 3.5–39 kg N_2_O ha^−1^) than those from other locations; exploratory analysis showed them to be outliers and these were considered to be unrepresentative of the study area.

Following the above processing, the data was reduced to 379 mean observations derived across N rate, source, timing and placement and their combinations. This final dataset consisted of 94 mean observations that focused exclusively on N application rate derived from 12 side-by-side experiments (≥3 N rates, including controls), 94 mean observations that originated from 8 side-by-side comparisons of N source alone, and the remaining observations consisted of N rate and N source in various combinations with N timing and N placement. Across these N management systems, 163 observations were derived from experiments conducted under irrigated maize (Colorado, Minnesota, and Nebraska) and 216 observations were obtained from rainfed systems. The irrigation systems data from Colorado alone accounted for 80% of the observations for irrigated maize systems. Similarly, 37% of the data originated from experiments where a maize-soybean rotation was applied, and these were predominantly from Indiana and Minnesota.

### Nitrogen uptake parameter estimation

For studies that reported only grain N uptake, GNU (Zebarth et al., 2008; Roy et al., 2014), total above-ground N uptake (TNU) was estimated by dividing maize grain N by a factor of 0.64 which is considered to be the N harvest index (NHI: total N in the grain as a fraction of TNU; Witt et al., 1999) that has been very stable over time as maize hybrids have changed (Ciampitti and Vyn, 2012; Mueller and Vyn, 2016). Conversely, where only TNU data was available (Venterea et al., 2011; Drury et al., 2012; Omonode et al., 2015; Venterea and Coulter, 2015; Omonode et al., unpublished), GNU was calculated by multiplying TNU by a factor of 0.64. From the available N rate and N uptake data, we calculated NRE, net N balance, NB (in terms of GNU, TNU), and surplus N (SN) as follows:

N recovery efficiency; NRE (%) = $\frac{TNU_{N}-TNU_{0}}{\Delta_{Napplied}}*$ 100
Grain uptake-based net N balance;
NNB_(GNU)_ (kg ha^−1^) = (*N*_*F*_ + *N*_*M*_ + *N*_*Rot*_) − *GNU*
Total uptake-based net N balance;
NNB_(TNU)_ (kg ha^−1^) = (*N*_*F*_ + *N*_*M*_ + *N*_*Rot*_) − *TNU*
Surplus N, SN (kg ha^−1^) = *N*_*F*_ − *TNU*

where *TNU* is total (grain+biomass) N uptake: *TNU*_*N*_, is the *TNU* of N-fertilized plots, *TNU*_0_ is the *TNU* of unfertilized plots, and Δ_*Napplied*_ is the differential of N applied; *N*_*F*_ = applied fertilizer N (kg N ha^−1^); *N*_*M*_ (kg N ha^−1^) = recoverable manure N; *N*_*ROT*_ = N (kg N ha^−1^) attributed to rotation when maize followed a legume as recommended for the state/region (Kentucky, 28; Colorado, 28; Indiana, 50; Nebraska, 50; Minnesota, 30; and Quebec, 30 kg N ha^−1^). We note here that for site-years where maize followed maize, no N credit was given to the following corn crop. Similarly, no credit was given for soybean nodules and/or N fixation; studies showed that soybean nodules and fixation are not major determinants of the soybean N credit (Bergerou et al., 2004).

Before statistical analysis, the data for management that involved N rate (N rate alone, rate + source, and rate + timing) was further grouped by 50 kg intervals (0, 1–50, 51–100, 101–150, 151–200, 201–250, 251–300; *n* = 7) to reduce some of the associated variability. For the rate alone data, grouping the data resulted in the following number of observations (n) for the groups: 22, 1, 13, 19, 9, 18, 2 with the following corresponding means: 0, 45, 73, 132, 174, 221, and 270 kg N ha^−1^, respectively, for the groups.

### Statistical data analysis

First, the relationships between N application rate and N uptake, NRE, SN, NNB [NNB_(GNU)_, NNB_(TNU)_] and N_2_O were evaluated using single-factor regression models where N rate was considered the independent variable, and dependent variables consisted of N_2_O, TNU, GNU, NRE, SN, and NNB. These relationships were assessed using data sets from experiments where management involved only rate of N application with 3 or more N levels, and included a control (zero N). The latter analysis was conducted because contrasting linear and nonlinear relationships are often reported for the relationship between N rate and N_2_O, and little is known about the relationship between N rate, TNU and NRE in the context of seasonal N_2_O emissions.

The relationships between N_2_O (area- and yield-scaled) and N uptake parameters (TNU, NRE, NNB, and SN) in the context of multiple N management practices were also determined using single-factor regression models. In constructing these regression models, N_2_O was considered the response variable and TNU, NRE, NNB, and SN constituted the independent variables. All regression analyses utilized the data points averaged over replicates for a given site-year. For analyses that assessed the relationships under N rate management systems, both the individual observations and the grouped N rate data were used in separate regression analyses for comparison. Finally, the relative significance of the contribution of the independent variables to the total variability associated with N_2_O was estimated using multiple regression models. All analyses were performed using SAS statistical package (SAS Institute, 2013) by invocating the PROC REG and PROC NLIN statements, respectively, for linear and non-linear regression models. The strengths of the relationships were assessed by the value of the regression coefficient of determination (linear: *r*^2^; nonlinear: *R*^2^), and the regression model was considered significant at *P* < 0.05 level probability. Graphs that visualized these relationships were produced using SigmaPlot version 13, from Systat Software, Inc., San Jose, CA (www.systatsoftware.com).

## Results

### Data overview

Grouping N rate by 50 kg N intervals significantly improved *r*^2^ values by up to 60% but did not necessarily improve the statistical significance (*P*-values) of the relationship. However, for easy comparison, results for the relationships obtained using both the grouped and individual observations data are presented; models using the grouped data are presented in figures, and those derived using the individual observations were presented in tables. Similarly, we present figures for the relationships between N_2_O and NNB_(TNU/GNU)_ and SN for comparison even when the *r*^2^ and *P*-values were similar for both parameters in some instances.

Seasonal N_2_O, TNU, and NRE varied widely in distribution (data not shown) as would be expected of data aggregated across soil, management, and climate variations. Emission was generally lower for relatively drier Colorado compared to other locations. On average, cumulative N_2_O was about 47% (1.67 kg N ha^−1^) greater for rainfed maize compared to irrigated maize cropping systems (0.89 kg N ha^−1^), perhaps because the latter was dominated by data from Colorado. Nitrogen recovery efficiency values ranged from 6 to 147%, with a mean of 56%. The NRE values exceeding 100% in the data occurred at lower N rates (<90 kg N) when TNU was significantly greater than N applied (a common response in the Midwestern United States). The average NRE observed here was similar to that reported for Indiana (Burzaco et al., 2014), but was much greater than those reported for on-farm trials (Cassman et al., 2002). Both NNB and SN ranged widely from large negative to positive values, and averaged −19.9 and −35.9 for NNB_(*TNU*)_ and SN, respectively; the latter negative values indicated that maize's TNU often exceeded fertilizer N inputs into the system.

### Relationship between N rate and maize yield, recovery efficiency, and N_2_O emission

**Figure 1:**
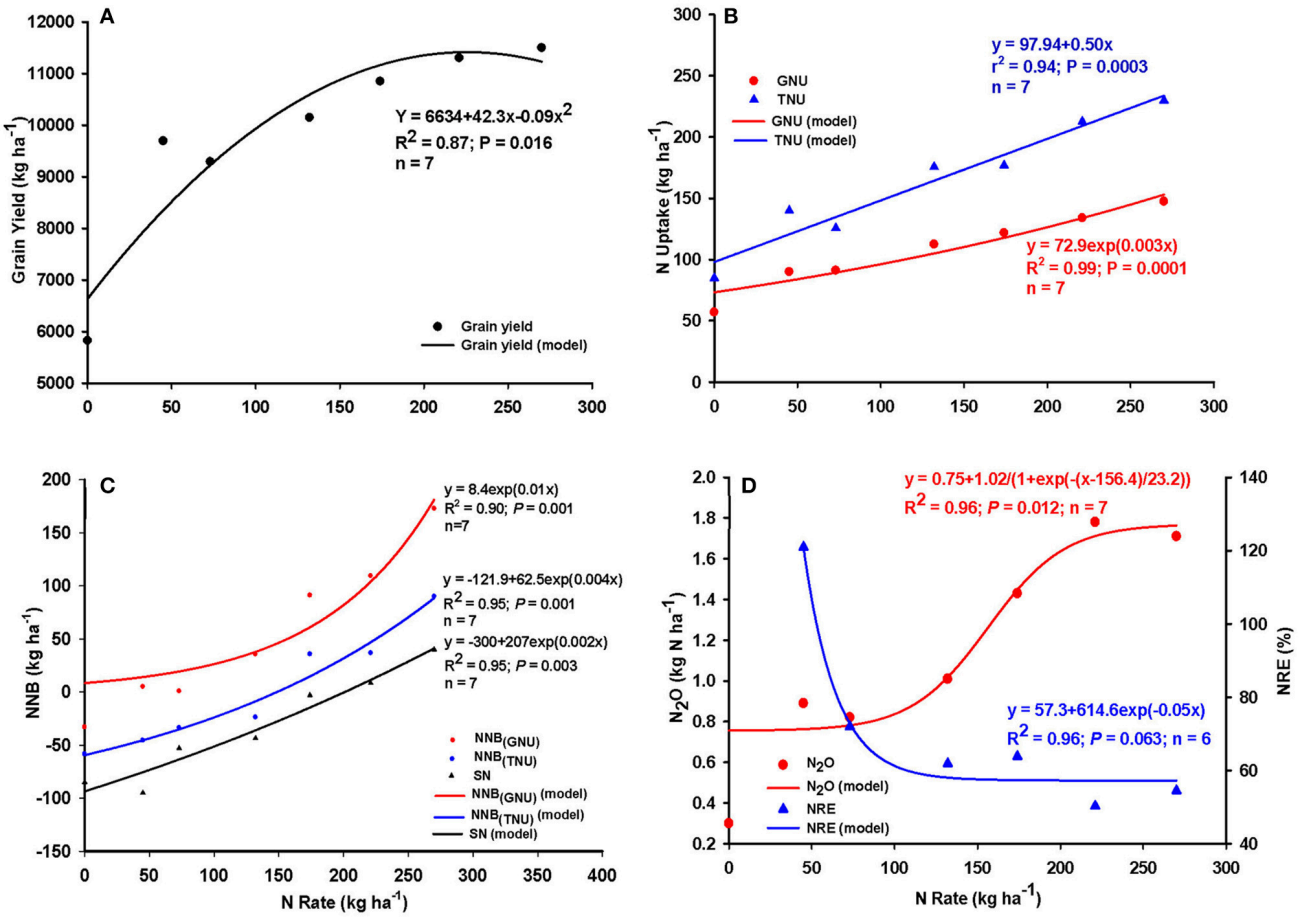
Relationship between N application rate and **(A)** maize grain yield **(B)** grain N (GNU) and total N uptake (TNU) **(C)** TNU, GNU, and surplus N (SN)-based net N balance (NNB), and **(D)** N recovery efficiency (NRE) and nitrous oxide emissions (N_2_O) across Colorado and Indiana (United States), and Quebec and Ontario (Canada) maize production systems. *n* = number of N rate groups, data points in x-axis represent mean value for the group.

The relationships between N application rate, maize yield, N uptake, NRE, NNB, SN, and seasonal N_2_O emissions based on the grouped N data are shown in Figure 1 while the relationships derived from the individual observations are shown in Table 1. A strong, non-linear relationship existed between the N rate and maize grain yield (Figure 1A: *R*^2^ = 0.87; individual observations: *r*^2^ = 0.71; *P* < 0.001; Table 1). Grain yield generally followed a quadratic model indicating that yield increased, on average, from about 6,600 kg ha^−1^ at zero N and plateaued at about 11,390 kg ha^−1^ at the 225 kg N ha^−1^ rate, beyond which grain yield was unlikely to respond to any additional increase of N application. Across these North America states or regions under consideration, N rate recommended for agronomic optimum maize yield ranges from about 150 kg N ha^−1^ for Minnesota to 220 kg N ha^−1^ for Indiana.

**Table 1:** Regression models and parameters for the relationships between N rate and grain yield, nitrous oxide (N_2_O) emission; nitrogen uptake (GNU, TNU), nitrogen recovery efficiency (NRE), net nitrogen balance (NNB), and surplus nitrogen (SN) based on the individual observations when management focused on N rate alone.

**Variables**	**Regression Parameters**				
	***N***	**Model**	**Equation**	***R*^2^**	***P < F***
Grain yield	84	Quadratic	*y* = 5993+44.4x−0.09x^2^	0.71	<0.001
N_2_O	84	Linear	*y* = 0.28+0.006x	0.43	<0.001
	84	Exponential	*y* = 0.47exp(0.006x)	0.40	<0.001
TNU	84	Linear	*y* = 88.4+0.57x	0.61	<0.001
	84	Exponential	*y* = 98.7exp(0.003x)	0.57	<0.001
GNU	84	Linear	*y* = 61.7+0.342x	0.65	<0.001
	84	Exponential	*y* = 67.71exp(0.003x)	0.62	<0.001
NRE	62	Linear	*y* = 83.98−0.14x	0.14	0.003
	62	Exponential	*y* = 88.5exp(−0.002x)	0.13	0.002
NNB_(*GNU*)_	80	Linear	*y* = −32.99+0.689x	0.82	<0.001
	80	Exponential	*y* = 7.5exp(0.013x)	0.71	<0.001
NNB_(*TNU*)_	80	Linear	*y* = −63.26+0.492x	0.59	<0.001
	80	Exponential	*y* = −107.5+49.6exp(0.005x)	0.61	<0.001
SN	84	Linear	*y* = −88.35+ 0.433x	0.47	<0.001
	84	Exponential	*y* = −124.2+42.2exp(0.005x)	0.49	<0.001

*N, Number of individual observations; GNU, grain nitrogen uptake; TNU, total nitrogen uptake*.

Similarly, strong and highly significant positive relationships existed between TNU and GNU and application rate (Figure 1B; relationships using point observations shown in Table 1). Overall, both linear and nonlinear models were good fits for the relationships between N rate and GNU (linear: *r*^2^ = 0.99) and TNU (linear: *r*^2^ = 0.94); GNU and TNU tended to increase linearly as N rate was increased up to about 225 kg N ha^−1^ (Figure 1B). Net N balance (NNB) in terms of TNU, GNU, and SN also generally increased as rate of N application was increased (Figure 1C). At zero or low N rates, N balance was close to zero (GNU), and negative for TNU and SN, but increased exponentially as N rate was increased above 150–200 kg N, especially for GNU. The rate of increase of N balance due to increase of N rate was in the order NNB_(GNU)_ > NNB_(TNU)_ > SN.

The relationships between N rates and NRE as well as seasonal N_2_O emission are shown in Figure 1D. The relationship between N rate and N_2_O emission appeared to be complex and was best described by a sigmoidal model (*R*^2^ = 0.96, *P* = 0.014) whereby the rate of increase of N_2_O was relatively small for N rates up 100–130 kg ha^−1^, followed by a sharp increase at N rates from 130 to 220 kg ha^−1^, and then leveled off at N rates >220 kg N ha^−1^. However, both the linear and exponential models were also good fits to the data, albeit with reduced coefficients of determinations (linear: *R*^2^ = 0.91; *P* = 0.001; exponential: *R*^2^ = 0.85; *P* = 0.003; graphs not shown). Overall, our model suggested that seasonal N_2_O emission was likely to increase linearly by about 5 g N per kg of N applied for rates up to 220 kg ha^−1^ (maize yield tend to plateau at 180–200 kg N ha^−1^ across North America as indicated in Figure 1A). In contrast, a significant (*P* < 0.005; *R*^2^ = 0.96) negative relationship existed between N rate and NRE (Figure 1D). Our model showed that maximum NRE occurred at low N rates of < 60 kg N ha^−1^, NRE decreased rapidly to about 60% as N rates progressed to 100 kg N ha^−1^, decreased less rapidly for rates between 130 and 150 kg N ha^−1^, and subsequently leveled for N rates exceeding 150 kg N ha^−1^. It is noteworthy that N_2_O loss increased rapidly in the 130–200 kg ha^−1^ N rate range, the same range of N applications at which NRE levels plateaued in these experiments.

We noted that in some locations, NRE exceeded the maximum attainable 100% (especially at lower N rates of 50–90 kg N ha^−1^) and that these extremes will impact the regression parameters. However, we were not justified in excluding NRE values exceeding 100% as these are typical for highly productive US Corn Belt and Canadian maize producing soils at lower N rates, and are indicative of N sources other than applied fertilizer N becoming more available to the maize plants when low N rates are applied (Abalos et al., 2016). For comparison, elimination of NRE values >100% resulted in the regression model: *y* = 67.4 − 0.04x (*r*^2^ = 0.66) which suggested that a NRE of about 64% would be expected at N rates of about 80 kg N ha^−1^, and that NRE would decrease by about 0.04% for every kg of additional N (data not shown).

### Relationship between nitrous oxide emission, nitrogen recovery efficiency, and net nitrogen balance under different N management practices

#### Nitrogen rate

**Figure 2:**
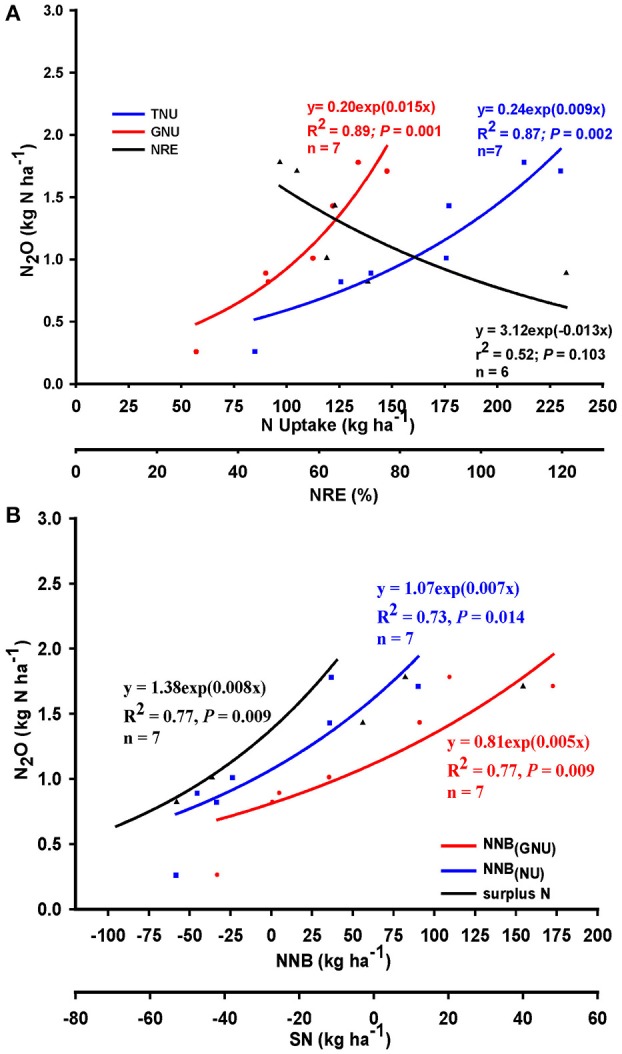
Relationships between nitrous oxide (N_2_O) emission and **(A)** N uptake and recovery efficiency (NRE), **(B)** net N balance (NNB) relative to TNU, GNU, and SN, when management focused on application rate across Colorado and Indiana (United States), and Quebec and Ontario (Canada) maize production systems; *n* = number of N rate groups, data points in x-axis represent mean value for the group.

The relationship between seasonal N_2_O emission, N uptake and recovery efficiency, and N balance when management focused only on N application rate across Colorado, Indiana, Ontario and Quebec are shown in Figure 2. A significant and positive relationship existed between seasonal N_2_O and N uptake (Figure 2A: TNU: *R*^2^ = 0.87, *P* = 0.002; GNU: *R*^2^ = 0.89, *P* = 0.001) which is contrary to the expectation that an increase of N uptake would result in a decrease of N_2_O emission. The model indicated that about 15 g N was likely to be emitted for every kg N taken up by the corn grain—about 40% greater than N emitted per kg of TNU. However, the relationship between N_2_O and NRE was negative (Figure 2A: *R*^2^ = 0.52, *P* = 0.103), and thus indicated that seasonal N_2_O emission was likely to decline as NRE was increased. Overall, the model indicated that N_2_O emission would decrease by up to 13 g ha^−1^ for every percentage point increase of NRE when NRE exceeded 100% at lower N rates, and by up to 40 g ha^−1^ for NRE < 80% when N rate exceeded ~150 kg N ha^−1^ (graph not shown). Using the individual observations for this analysis changed neither the direction nor the statistical significance of these relationships even as the strength of the relationship was significantly reduced (Table 2: GNU: *r*^2^ = 0.22, *P* = <0.001; TNU: *r*^2^ = 0.18, *P* = <0.001; NRE: *r*^2^ = 0.14, *P* = 0.003).

**Table 2:** Regression models and parameters for the relationships between nitrous oxide (N_2_O) emission and nitrogen uptake (GNU, TNU), nitrogen recovery efficiency (NRE), net nitrogen balance (NNB), and surplus nitrogen (SN) based on the individual observations when management focused on N rate alone.

**Variables**	**Regression Parameters**				
	***N***	**Model**	**Equation**	***R*^2^**	***P < F***
GNU	84	Linear	*y* = −0.065+0.011x	0.215	<0.0001
	84	Exponential	*y* = 0.430exp(0.008x)	0.172	<0.0001
TNU	84	Linear	*y* = 0.147+0.006x	0.178	<0.0001
	84	Exponential	*y* = 0.459exp(0.005x)	0.162	<0.0001
NRE	62	Linear	*y* = 2.064−0.013x	0.136	0.0031
	62	Exponential	*y* = 2.414exp(−0.011x)	0.135	0.0033
NNB_(*GNU*)_	80	Linear	*y* = 0.676+0.008x	0.328	<0.0001
	80	Exponential	*y* = 0.706exp(0.007x)	0.297	<0.0001
NNB_(*TNU*)_	78	Linear	*y* = 1.080+0.008x	0.244	<0.0001
	78	Exponential	*y* = 1.0066exp(0.007x)	0.228	<0.0001
SN	84	Linear	*y* = 1.350+0.009x	0.311	<0.0001
	84	Exponential	*y* = 1.263exp(0.008x)	0.308	<0.0001

*N, Number of individual observations; GNU, grain nitrogen uptake; TNU, total nitrogen uptake*.

Similarly, under N rate management, relatively strong and positive relationships existed between N_2_O emission and NNB [NNB_(TNU)_, NNB_(GNU)_, SN] such that emission increased as N balance was increased, albeit with sensitivity that decreased in the order: SN > TNU > GNU (Figure 2B). At negative NNB, N_2_O emission was generally small (about 0.6 kg N ha^−1^) but increased linearly as NNB approached 0 kg N, and then exponentially to about 2.0 kg N_2_O-N ha^−1^ as NNB increased to about 40, 75, and 175 kg N, respectively, for SN, TNU, and GNU (compared to the 40–50 kg SN thresholds reported by Van Groenigen et al., 2010; Venterea et al., 2016). Overall, the model suggested that N_2_O emission will increase by about 8, 7, and 5 g N ha^−1^, respectively, for every kg increase of SN, TNU, and GNU.

#### Nitrogen rate and source

Relationships between N_2_O and TNU, NRE, and NNB_(TNU)_ when management treatments involved both N rate (ranging from 0 to 220 kg N ha^−1^) and source combination (urea ammonium-nitrate (UAN) alone and UAN+nitrapyrin) in experiments conducted in Indiana are shown in Table 3. No measurable and/or consistent relationship was found between N_2_O and TNU when management choices involved N rate+source combinations. On the contrary, the rate+source combination tended to confound the relationship between N_2_O and NRE, such that a fairly strong positive linear relationship existed between N_2_O and NRE (*r*^2^ = 0.21), perhaps driven by addition of a nitrification inhibitor (nitrapyrin) to a common N fertilizer source (UAN). However, like management by N rate alone, relationships between N_2_O and NNB_(TNU)_ (*r*^2^ = 0.34; *P* = 0.006) and SN (data not shown) were fairly strong and positive even though both relationships also appeared to be influenced by nitrapyrin application.

**Table 3:** Linear regression parameters for the relationships between nitrous oxide (N_2_O) emission and TNU, NRE and NNB when management focused on rate and source, rate and timing, and rate, source and timing combinations, based on individual observations.

**Nitrogen management**	**TNU**				**NRE**				**NNB**_**(TNU)**_			
	***N***	**Reg. model**	***R*^2^**	***P* < *F***	***N***	**Reg. model**	***R*^2^**	***F* < *F***	***N***	**Reg. model**	***R*^2^**	***P* < *F***
Rate and source	20	*y* = −0.00x+1.47	0.00	0.972	16	*y* = 0.032x−0.09	0.21	0.076	20	*y* = 0.015x+1.52	0.34	0.006
Rate and timing	30	*y* = −0.003x+2.13	0.01	0.552	26	*y* = −0.017x+2.71	0.33	0.002	30	*y* = 0.009x+2.13	0.26	0.004
Rate, source and timing	24	*y* = 0.021x−1.17	0.46	0.001	16	*y* = 0.024x+0.97	0.14	0.144	24	*y* = 0.014x+1.86	0.14	0.066

*N, Number of individual points; TNU, total nitrogen uptake; NRE, N recovery efficiency; NNB, net N balance. Data for rate+source from experiments conducted in Indiana, USA; rate+timing data from experiments conducted in Indiana and Minnesota (USA) and New Brunswick, Canada; and rate+source+timing data obtained from experiments in Indiana, USA*.

#### Nitrogen rate and timing

**Figure 3:**
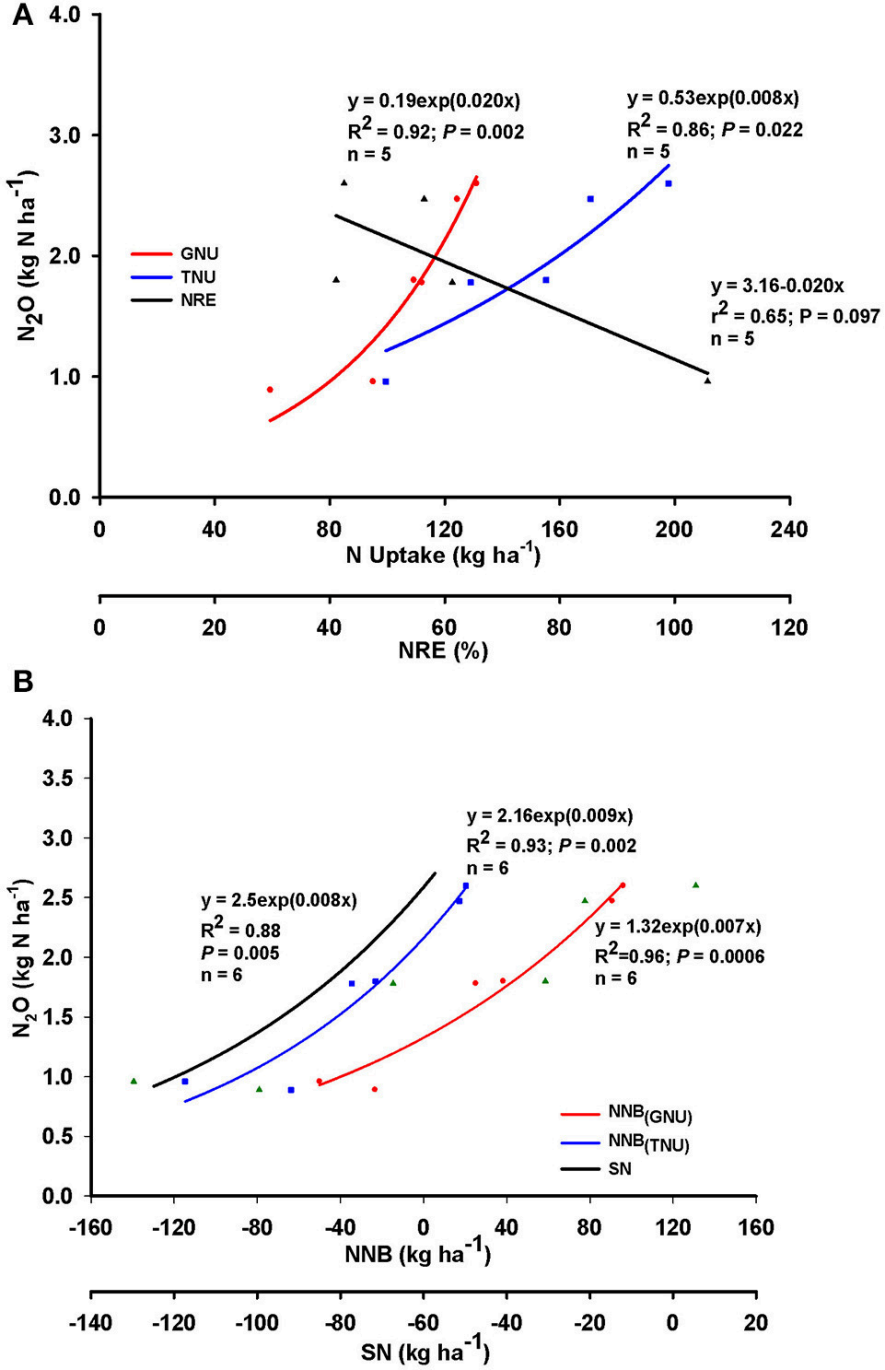
Relationship between nitrous oxide (N_2_O) emission and **(A)** grain N (GNU) and total N uptake (TNU) and N recovery efficiency (NRE), and **(B)** net N balance (NNB) relative to TNU, GNU and surplus N (SN) when management focused on N application rate and timing combination across Indiana and Minnesota (United States), and New Brunswick (Canada). *n* = number of N rate groups and points within each n group are mean values of 12, 4, 14, 8, 12, and 4 observations, respectively.

Data used to assess the relationships between N_2_O, N uptake and use efficiency, and N balance when management consisted of N rate+timing combination were obtained from experiments conducted in Indiana and New Brunswick, Canada (preplant and common/regular sidedress at maize growth stage V6; hereafter referred to simply as “sidedress”), and Minnesota (common/regular sidedress at V6 and late-split sidedress at maize growth stage V14; hereafter referred to, respectively, as “early” and “late sidedress”). Rate+timing combination management resulted in similar directions for the relationships between N_2_O and N uptake, NRE, and NNB as with management by N rate alone (Figure 3). However, compared to N rate, N_2_O increased as N uptake was increased at similar rate for TNU (model slope = 0.008; Figure 3A), but at a relatively greater rate for GNU (model slope = 0.020; N rate = 0.015). Similarly, a negative relationship occurred between N_2_O and NRE under rate+timing combination (Figure 3A); N_2_O was reduced by about 20 g N per % NRE compared to the 13 g N per % NRE for N rate alone. Under rate+timing combinations, highly significant positive relationships existed between N_2_O and NNB_(GNU)_, NNB_(TNU)_ and SN; rate of N_2_O increase per kg of N balance was in the order: NNB_(GNU)_ < SN < NNB_(TNU)_ (Figure 3B). Overall, N_2_O loss increased by 7, 8, 9 g N, respectively, per kg of NNB_(GNU)_, SN, and NNB_(TNU)_. This indicated that a greater opportunity existed for rate+timing practices, compared to N rate alone, to reduce N_2_O emission by increasing grain N uptake.

**Figure 4:**
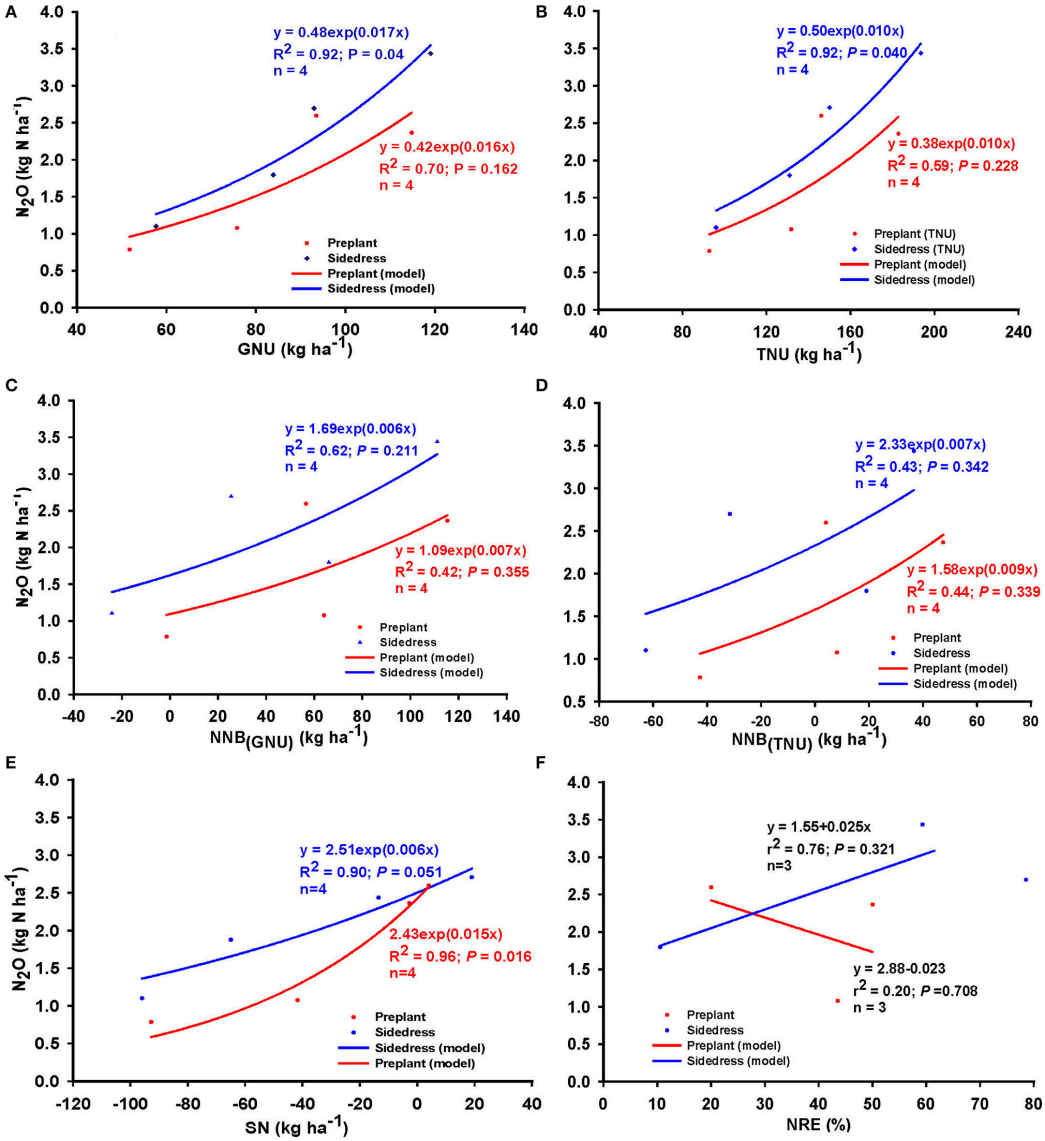
Relationship between nitrous oxide (N_2_O) emission and **(A)** grain N uptake (GNU), **(B)** total N uptake (TNU), net N balance for **(C)** grain N [NNB_(GNU)_] **(D)** total N uptake [NNB_(TNU)_] and **(E)** surplus N (SN), and **(F)** N recovery efficiency (NRE) when N was applied preplant/at planting and early sidedress (V6) across Indiana (United States) and New Brunswick (Canada). *n* = number of N rate groups, and points within each n group are mean values of 4, 4, 2, and 4 observations, respectively.

Timing (preplant/at-planting vs. regular sidedress; averaged of N rates) had variable effects on the relationships between N_2_O emission and N uptake, NRE, and NNB (Figure 4). For both at-planting and regular sidedress timings, the relationships between N_2_O and N uptake and net N balance were positive and relatively strong (*R*^2^ ranged from 42 to 92%), but seasonal N_2_O emission was generally greater for sidedress than for at-planting application timing (Figures 4A–E). Under sidedress timing, rate of N_2_O emitted per kg GNU was about 10% greater than for at-planting (Figure 4A). That trend was reversed for NNB_(GNU)_ and NNB_(TNU)_ where the rate of N_2_O emission was greater per kg increase of N balance under at-planting application (Figures 4C,D). However, timing effects on N_2_O emission were more dramatic for SN; rate of N_2_O increased by 15g N per kg of SN for at-planting but by only 6g N per kg of SN under sidedress application. Timing influence on the relationship between N_2_O and NRE was mixed; positive under sidedress, and negative for at-planting (Figure 4F). Overall, N_2_O was reduced by 23 g N per 1.0% NRE increase for at-plant application, and increased by 25g N per 1.0% NRE gain with sidedress application.

**Figure 5:**
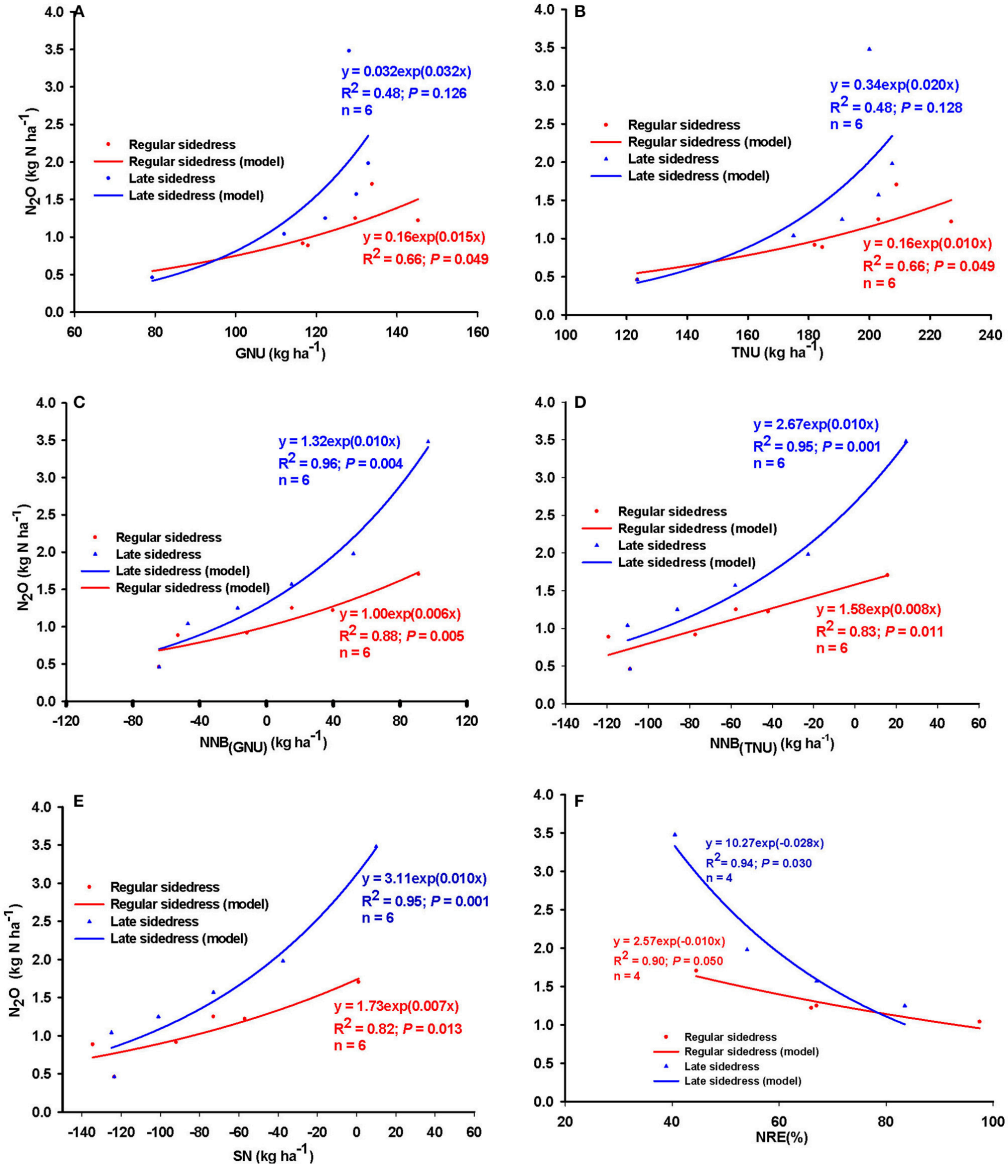
Relationships between nitrous oxide (N_2_O) emission and **(A)** grain N (GNU) and **(B)** total N uptake (TNU), net N balance for **(C)** grain N [NNB_(GNU)_], **(D)** total N uptake [NNB_(TNU)_] and **(E)** surplus N (SN), and **(F)** N recovery efficiency (NRE) when N was applied at early/regular sidedress (V6) and late sidedress (V14) in Minnesota. *n* = number of N rate groups, and points within each n group are mean values of 2 observations.

The relationships between N_2_O and N uptake and NNB under regular and late application timings are shown in Figures 5A–E. Late sidedress increased N_2_O by 20–30 g N per kg increase of N uptake which was about 50% greater than the regular sidedress timing (Figures 5A,B). Both the regular and late timings resulted in significant positive relationships between N_2_O emission and NNB_(GNU)_, NNB_(TNU)_, and SN (Figures 5C–E). However, N_2_O emitted per kg increase of NNB_(TNU)_, NNB_(GNU)_, and SN was generally greater for late sidedress relative to the regular sidedress. Under late sidedress timing, about 10 g N_2_O was emitted per kg increase of NNB_(GNU)_,NNB_(TNU)_, and SN which was 40, 20, and 30%, respectively, greater than under regular sidedress application. Similarly, a negative relationship existed between N_2_O and NRE, regardless of whether timing was regular or late sidedress (Figure 5F). However, late sidedress increased the strength of the relationship between N_2_O and NRE by 42, 29, and 74%, respectively, relative to N rate alone, rate+timing, and preplant timing N application management options. Overall, our models suggested that at low NRE of 40–50%, late sidedress timing will result in greater N_2_O emission, but rates of emission will dramatically decrease as NRE increased up to about 80%. The model showed that N_2_O decreased by 10 g N ha^−1^ per 1.0% improvement of NRE under regular sidedress, and by about 28 g N ha^−1^ per 1.0% increase of NRE under late sidedress timing (Figure 5F).

#### Nitrogen rate, source, and timing

Limited data from Indiana where N rate and source (UAN with and without nitrapyrin) and timing (preplant vs. sidedress) treatment were applied simultaneously indicated that the rate+source+timing combination confounded the relationships between N_2_O and NRE and NNB, perhaps due to the dominant effect of N source (Table 3). When N source and timing influences were separated, the relationship between N_2_O and NRE was negative linear (i.e., N_2_O emission decreased as NRE increased) for timing, but was strongly positive linear for N source, especially under UAN+nitrapyrin (*r*^2^ = 0.74) compared to UAN alone (*r*^2^ = 0.20, data not shown).

#### Nitrogen source, source and placement, and timing combinations

The dataset used to evaluate N source influence on the relationships between N_2_O and N uptake, use efficiency and balance were obtained from experiments where management involved either N source alone (urea, UAN, AgrotainPlus®, UAN+AgrotainPlus®; polymer coated urea, ESN®; and stabilized urea—SUPERU®) in Colorado, Kentucky, Minnesota, and Ontario, or in combination with timing (Indiana; UAN/UAN+nitrapyrin applied at-planting or sidedress), or placement (Colorado and Minnesota; broadcast and banded). Our analysis indicated that N source alone had inconsistent, albeit confounding, influences on the relationships between N_2_O and TNU, NRE and NNB (*r*^2^: 0.01–0.21, Table 4). Similarly, source+placement combination or placement (averaged over N sources) resulted in inconsistent relationships between N_2_O and TNU, NRE and NNB (data not shown).

**Table 4:** Regression parameters for the relationships between nitrous oxide (N_2_O) emission and TNU, NRE and net N balance when N fertilizer sources involved urea, ESN, and stabilized N (SUPERU, AgrotainPlus).

***N* Source**	**TNU**				**NRE**				**NNB**_**(TNU)**_			
	***N***	**Reg. model**	***R*^2^**	***P* < *F***	***N***	**Reg. model**	***R*^2^**	***P* < *F***	***N***	**Reg. model**	***R*^2^**	***P* < *F***
Urea	32	*y* = 0.001x+1.63	0.00	0.81	20	*y* = 0.012x+0.54	0.14	0.10	32	*y* = −0.007x+1.90	0.11	0.06
ESN	30	*y* = −0.002x+2.62	0.00	0.87	18	*y* = 0.029x−0.11	0.21	0.06	30	*y* = −0.016x+2.26	0.10	0.08
Stabilized N	23	*y* = 0.005x−0.02	0.03	0.40	23	*y* = 0.012x+0.28	0.07	0.62	23	*y* = −0.006x+0.94	0.15	0.07
Across N sources	61	*y* = 0.001x+1.56	0.00	0.84	61	*y* = 0.021x+0.12	0.14	0.01	85	*y* = −0.011x+1.79	0.09	0.01

*ESN, Environmentally smart nitrogen (polymer-coated urea; 44-0-0); stabilized N, nitrification+urease inhibitors: Agrotain with urea (46-0-0), AgrotainPlus with urea ammonium-nitrate (28-0-0), superU (urea 46-0-0). N, number of observations; TNU, total nitrogen uptake; NRE, N recovery efficiency; NNB_(TNU)_, net nitrogen balance*.

**Figure 6:**
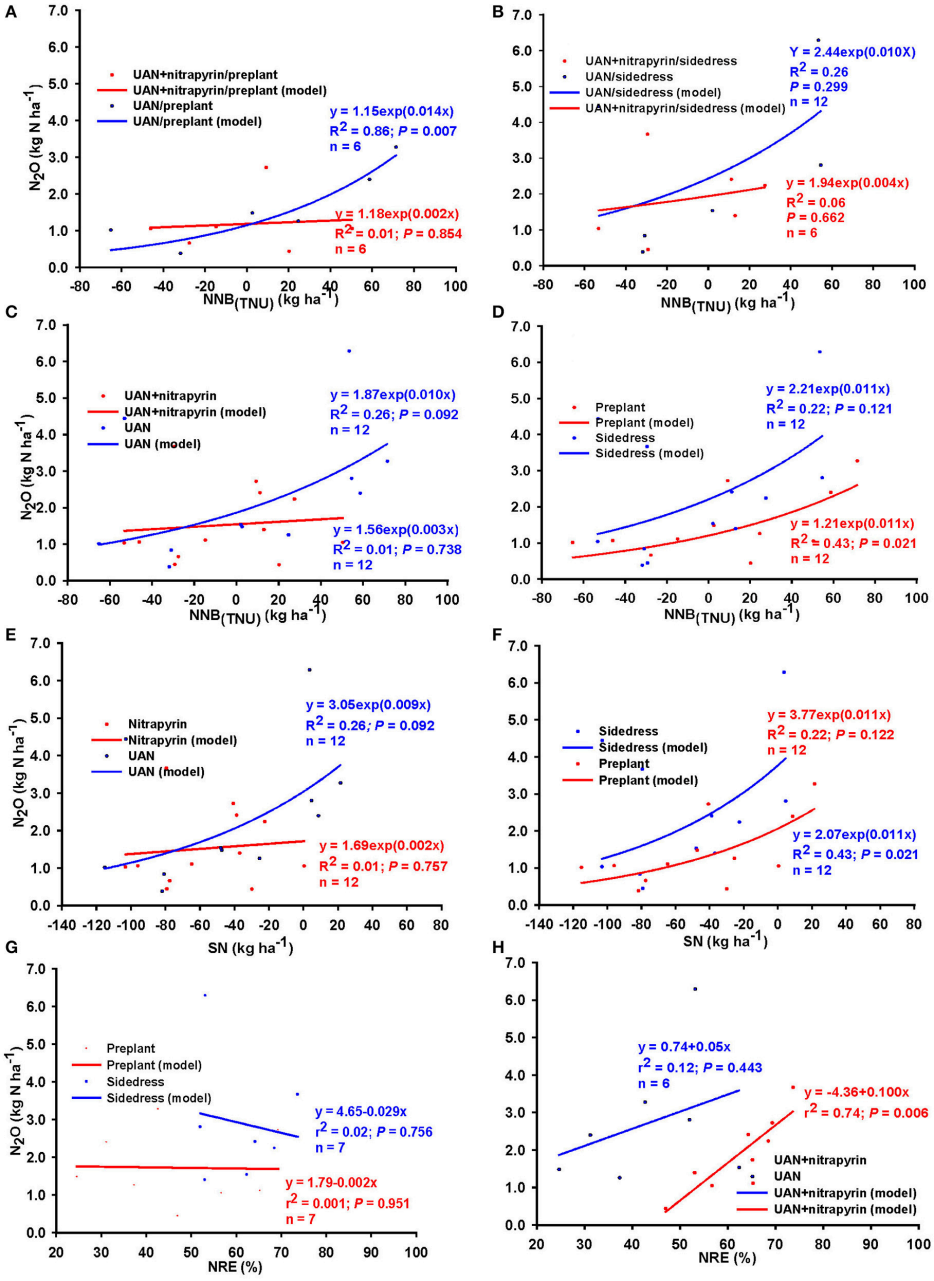
Relationship between N_2_O emission and N balance [NNB_(TNU)_] due to N source and timing combination applied at **(A)** preplant, **(B)** sidedress; **(C)** due to N source averaged over timing, **(D)** due to timing averaged over source; relationship between N_2_O and surplus N **(E)** averaged over timing, **(F)** averaged over N sources; and relationship between N_2_O and NRE **(G)** averaged over N source, and **(H)** averaged over timing, across Indiana (United States). *N* = number of observations; for **(A,B)**, *n* = number of N rate groups and consisted of mean values of 2 observations.

Nitrogen source (UAN, UAN+nitrapyrin) and timing (preplant or sidedress) combination effects on these relationships were variable (Figure 6). In general, source+timing combination resulted in relatively stronger relationships when N source was UAN, and seasonal N_2_O was greater for UAN relative to UAN+nitrapyrin, regardless of timing of application (Figures 6A,B). Averaged over timing, the relationship between N_2_O and NNB_(TNU)_ and SN were similar; N_2_O was generally greater with UAN relative to UAN+nitrapyrin; N_2_O increased by 10 g N per kg NNB but no measurable change was observed for UAN+nitrapyrin (Figures 6C–F). Across N sources, N_2_O increased as NNB was increased for both timings, but at a higher rate for sidedress application as N balance exceeded zero.

Similarly, N source and timing had variable effects on the relationship between N_2_O and NRE (Figures 6G,H). Averaged across timing, positive linear relationships between N_2_O and NRE were found perhaps due to the confounding effect of the N sources. In contrast, timing resulted in negative relationships between N_2_O and NRE such that N_2_O decreased by up to 29 g per 1.0% NRE gain under sidedress and about 2 g per 1.0% NRE gain for preplant application.

### Relative effect of nitrogen recovery efficiency and balance on N_2_O emission

We quantified the relative influence of the N management practices on the relationships between N_2_O and N uptake parameters using multiple regression models. These models showed that N_2_O emission was most associated with these variables when management focused solely on N application rate, but the strength of the relationship progressively decreased in the order: rate > timing > source. When management focused only on N rate, the multiple regression model showed that all the variables associated with N uptake and N balance accounted for 40% of the variability associated with N_2_O emissions. However, NNB_(TNU)_ and NNB_(GNU)_alone accounted for 32 and 3%, respectively, of the total variability as represented by the model:

$${\text{N}}_{2}\text{O}=0.99+0.006{\text{NNB}}_{\left(\text{TNU}\right)}+0.004{\text{NNB}}_{\left(\text{GNU}\right)};P=0.0001.$$

When management focused on N source, NRE was the dominant factor and accounted for 12 of the about 18% variability associated with N_2_O emission, but no variable had a dominant influence when management involved N source plus timing and/or placement. Across all management systems, the multiple regression model indicated a rather weak functional linear relationship between these variables and N_2_O emissions (*r*^2^ = 0.046; *P* = 0.034) which perhaps underscored the effect of variability associated with the dataset. Nevertheless, the step-wise regression model showed that NNB_(GNU)_ and SN accounted for about 78% of the total variability associated with N_2_O as follows:

$${\text{N}}_{2}\text{O}=0.81+0.008{\text{NNB}}_{\left(\text{GNU}\right)}-0.004\text{SN};P=0.008.$$

## Discussion

### Nitrogen rate, and rate and timing combinations

The strong positive relationship between N application rate and N_2_O emissions reported here affirmed the field-level findings of several authors who reported either a strong positive linear relationship between N application rate and N_2_O (MacKenzie et al., 1998; Gagnon et al., 2011; Roy et al., 2014), or a non-linear relationship (Breitenbeck and Bremner, 1986; McSwiney and Robertson, 2005), or both, depending on timing (Venterea and Coulter, 2015). Several meta-analyses also found strong positive relationships between N application rate and seasonal N_2_O emissions (Eichner, 1990; Van Groenigen et al., 2010; Decock, 2014). However, in this study, the relationship between N_2_O and N rate was sigmoidal in nature and N_2_O emission tended to rise sharply for N rates of 130–220 kg N ha^−1^ (Figure 1D) that are recommended for this region, which did not support earlier suggestions by these previous authors that N application at recommended rate was likely to decrease N_2_O emissions. Perhaps a reduction of N application rate within the 150–220 kg N ha^−1^ rate window by an amount that does not significantly reduce yield (Figure 1A), but is enough to increase NRE, is a better option. This study showed that the indirect mechanism underlying such a response is perhaps because NRE tended to be greater at relatively lower N rates.

To the best of our knowledge, very few studies (whether individual experiment or meta-analysis) have reported relationships between seasonal N_2_O emission and maize N uptake and N balance; therefore, comparing our results with the existing literature was rather difficult. Overall, the positive relationship between N_2_O and N uptake observed here was contrary to the general expectation that an increase of N uptake will result in N_2_O emission reduction. However, this positive relationship was similar to several studies where N management practices that achieved the highest recovery of fertilizer N in crop biomass also resulted in the highest N_2_O emissions (Fujinuma et al., 2011; Gagnon et al., 2011). Our analysis over a multitude of N rates and environments showed that NRE dramatically decreased as N rate was increased, which conceivably made more N available for denitrification even as TNU increased.

Negative relationships between N_2_O and NRE, especially when management involved rate of N application, as also reported by Van Groenigen et al. (2010) in a meta-analysis (even though these authors aggregated NRE, referred to as NUE, across different crop species), affirmed the hypothesis that an increase of NRE would result in a decrease of N_2_O emissions. However, the less-than-perfect but significant relationship of N_2_O emission to NRE reported for this study was probably because the periods of peak N_2_O emission and plant recovery of applied N during the growing season hardly coincided, or because our analysis involved N_2_O as the only source of N loss from these systems, or both. For most of North America, about 50–80% of seasonal cumulative N_2_O emissions occur in 30–40 days following N application when the maize plant is at around V6 and plant N uptake is still relatively small (Omonode et al., 2011; Halvorson and Del Grosso, 2012). Several studies also found that N_2_O accounted for only a small fraction of the total N that are lost from the system following N application; most N losses occurred as ammonia volatilization and/or nitrate leaching which, respectively, accounted for about 10 and 30% of applied N compared to about 3% for N_2_O losses (Mosier et al., 1998; De Klein et al., 2006; Venterea et al., 2012). Similarly, the relatively strong positive relationships between N_2_O emission and N balance in relation to NNB_(TNU)_, NNB_(GNU)_, and SN observed here were consistent with Van Groenigen et al. (2010) and Venterea et al. (2016).

Furthermore, our finding of strong influence of timing (in combination with N rate or source) on the relationships of N_2_O to N balance and SN was generally similar to recent reports by Venterea et al. (2016). Our analysis showed that these relationships were stronger, and N_2_O emissions greater, under regular sidedress timing compared to preplant/at planting, and for late sidedress relative to regular sidedress. Overall, the rate of N_2_O increase per kg of N balance was greatest for SN and smallest for NNB_(GNU)_, especially for late sidedress application. However, at recommended N rates (150–220 kg ha^−1^), N balance was several orders of magnitude greater for NNB_(GNU)_ (70–175 kg ha^−1^ under N rate, and 50–100 kg ha^−1^ under rate and timing combination) compared to near 0 kg for SN. Therefore, greater N_2_O emission rate for SN relative to NNB_(GNU)_ was probably because SN from regular or late sidedress N application resulted in a greater short-term availability of N for denitrification, even as crop N uptake rate was expected to be greater at the V14 growth stage (Venterea and Coulter, 2015). However, the magnitude of NNB_(GNU)_ meant there is great potential for significant amounts of N_2_O to be emitted later in the cropping season if available mineral N was not taken up by the maize plant. Studies have shown that grain N uptake was a major parameter that have changed over time (and across hybrids era), and grain N tended to be mostly associated with new plant N uptake during the grain filling period in modern hybrids (Ciampitti and Vyn, 2013). Thus, practices that improve grain N uptake by synchronizing availability with uptake are likely to reduce overall N losses through volatilization or leaching, and ultimately reduce seasonal N_2_O emission in maize.

### N source, source and placement, and timing combinations

The published experiments from which the dataset used to examine the influence of N source on the relationships between N_2_O and the N uptake dynamics showed that ESN, stabilized urea (SUPERU, AgrotainPlus) and nitrapyrin-amended UAN by themselves reduced N_2_O emissions, relative to conventional urea and UAN within the location in which they were tested (Halvorson et al., 2010a,b, 2011, 2016a; Venterea et al., 2011; Drury et al., 2012; Omonode and Vyn, 2013; Burzaco et al., 2014; Decock, 2014). Our analysis of variance that used the plot-level observations aggregated over site-years for each of these locations showed that these N sources significantly affected N_2_O emissions and fertilizer induced emission factor (FIEF) but had no effect on NRE and NNB_(TNU)_, regardless of location (Table 5). Overall, N_2_O loss was greater for the conventional urea and UAN, and emission was reduced by 19–48% when nitrification and/or urease inhibitors were added to those N sources. Therefore, the rather confounding influence of N source on the relationships between N_2_O and NRE, and N balance observed here suggested that N source by itself had little and/or variable effect on NRE and its relationship to N_2_O emission. Table 5 showed that NRE and NNB_(TNU)_ were similar for all the N sources and suggested that some of the N made available from ESN, stabilized urea and nitrapyrin-amended UAN later in the growing season, following initial delay of nitrification of applied N, were probably leached out of the rooting zone and not recovered by maize plants. Therefore, the observed difference of N_2_O between ESN, AgrotainPlus and nitrapyrin-amended UAN relative to UAN or urea probably resulted from high emission peaks that usually occur early in the season following fertilizer application at early maize developmental stages (Omonode et al., 2011; Halvorson and Del Grosso, 2012).

**Table 5:** Nitrogen source effects on seasonal nitrous oxide emission, nitrogen recovery efficiency (NRE), net nitrogen balance [NNB_(TNU)_], fertilizer induced emission factor (FIEF), and emission reduction (ER) in selected locations across North America.

**Location**	***N* Source**	□ **Nitrous oxide and N uptake parameters**				
		**N_2_O (kg N ha^−1^)**	**NRE (%)**	**NNB_(TNU)_ (kg ha^−1^)**	**FIEF (%)**	**ER (%)**
Colorado	ESN	0.92b	53.9a	23.2a	0.36b	19.3
	Stabilized urea§	0.59c	56.3a	11.86a	0.21bc	48.2, 20.3
	UAN	0.74bc	50.7a	19.3a	0.29b	.
	Urea	1.14a	54.3a	21.52a	0.45a	.
Indiana (rainfed)	UAN	2.35a	52.7 a	19.8a	1.23a	.
	UAN+nitrapyrin	1.69b	57.6a	9.1 a	0.58b	28.1
Minnesota (rainfed, irrigated)	ESN	1.17ab	48.1a	−21.6a	0.44ab	19.9
	Stabilized urea	0.86b	52.7a	−27.6a	0.26b	41.1
	Urea	1.46a	52.2a	−25.4a	0.60a	.

□ *N_2_O and N uptake parameters data from Colorado consisted of 8 site -years (2007–2014) under irrigation; Indiana: 4 site-years (2010–2013) in rainfed condition; and Minnesota: 5 site-years (2008–2012) in rainfed and irrigated conditions. §Emission reduction for Colorado calculated relative to urea ammonium-nitrate (UAN) and urea, respectively; NNB_(TNU)_ = net nitrogen balance based on total N uptake. Within location and column, N_2_O and N uptake parameters followed by the same letters are not significantly different at P = 0.05*.

### Implication for management

Overall, the results from this analysis confirmed that relatively strong functional relationships existed between seasonal N_2_O emissions and N rate, N uptake (both GNU and TNU), and NRE (especially when N management involved appropriate timing of application). Clearly, N applications that exceeded recommended agronomic optimum N rates (ranging from about 150 kg in Minnesota to 220 kg N ha^−1^ in Indiana) may increase TNU, but will result in reduced NRE especially in the US Corn Belt where intrinsic soil N is relatively high. Similarly, our results established that N rate and timing of application were critically important N management combinations that have the potential to influence both TNU and NRE, and their relationships to N_2_O emission. Thus, application of the appropriate amount of N at the right time, especially at planting and/or early sidedress timings, was more likely to reduce N_2_O emissions relative to split applications involving sidedressing at >V12 maize growth stage. However, given the large inherent variability associated with the dataset due to differences in soil, climatic conditions, and management factors, the actual size of the impact associated with N rate and timing will vary for different agroecological and/or cropping systems. The model would need to be validated to be agroecology-specific.

While N sources by themselves have been shown to result in N_2_O emission differences (Halvorson et al., 2010a,b, 2011; Halvorson and Del Grosso, 2012; Burzaco et al., 2014; Vyn et al., 2016), our analyses clearly indicated that N source effect on N_2_O emission was not directly related to their effect on NRE. On the contrary, N source tended to confound the N_2_O versus NRE relationship; therefore, further research on N source-specific models are needed to better understand the relationship between NRE and N_2_O when N management is focused on N source.

Overall, we found the strongest and most consistent relationship between cumulative N_2_O and N balance (whether GNU or TNU). This was not entirely surprising because net N balance encompasses N availability in relation to both in-season fertilizer application, and the N that was carried over from the previous crop years due to practices such as rotation, cover crop, or manure application (i.e., the total size of N inputs). Thus, effective management to both improve NRE and reduce N_2_O must necessarily involve evaluating and adjusting for the N balance in the cropping system. However, our model suggested that management systems achieving a net N balance of <50–60 kg N ha^−1^ would both reduce the amount of N applied, and possibly ensure seasonal N_2_O would be reduced to a minimum.

We readily acknowledge that some of the results of this study may have been constrained by the structure of the dataset (e.g., limited number of observations for some N management combinations). Similarly, our N balance estimate did not include N derived from atmospheric deposition which can vary significantly with location. However, this analysis showed that a careful selection of integrated N management practices has the potential to maximize NRE and reduce seasonal N_2_O emissions. Although the numerical strength of the relationship between cumulative N_2_O and either TNU or NRE was relatively small for the N rate and timing combination, the relationship was statistically highly significant, and thus indicated that optimized N application rates and timing (especially at planting or at early sidedress) has the potential to both increase NRE and reduce N_2_O loss, regardless of N source. However, to further maximize the beneficial effects of rate-by-timing practices for N_2_O emission reductions, closer attention should be paid to the net N balance of the cropping system.

We strongly recommend that future N_2_O emissions studies incorporate a systems research approach involving N source, timing and/or placement interactions where crop N uptake and recovery efficiency parameters are also determined. This will enable scientists to better assess and understand the effects of these complex management practices on cereal grain N uptake and NRE and how they relate to N_2_O emissions.

## Author contributions

TV conceptualized the review and the pertinent relationships to explore, obtained the requisite funding, and helped write the paper. RO acquired the background data where necessary, conducted the statistical analyses and helped write the paper. AH provided background data from their prior publications, recommended scientific approaches to the data set to help guide the discussion, and helped to edit various drafts. BG helped with ideas and edits.

### Conflict of interest statement

The authors declare that the research was conducted in the absence of any commercial or financial relationships that could be construed as a potential conflict of interest. The reviewer EAM and handling Editor declared their shared affiliation, and the handling Editor states that the process met the standards of a fair and objective review.
