# Supplementary material for: Achieving Lower Nitrogen Balance and Higher Nitrogen Recovery Efficiency Reduces Nitrous Oxide Emissions in North America's Maize Cropping Systems
Source: Front Plant Sci. 2017 Jun 23;8:1080. doi: 10.3389/fpls.2017.01080 (PMC5481850; doi:10.3389/fpls.2017.01080)
Supplement: Supplementary file 1 [file Table1.docx]

**Supplemental Table 1**

Table S1. Data source, locations, site management systems, and treatment combinations that resulted in the data sets used in this analysis.

| Data Source | Location | Study Period | Water mgmt. | N Management Applied | | | |  | N Uptake | |
| --- | --- | --- | --- | --- | --- | --- | --- | --- | --- | --- |
|  |  |  |  | N Rate  (kg/ha) | Source | Timing | Placement |  | GNU | TNU |
| Adviento-Borbe et al. (2007) | NE/USA | 2001-2005 | Irrigation | 130-310 | - | - | - |  | yes | Yes |
| Burzaco et al. (2014) | IN/USA | 2010-2011 | Rainfed | 0-180 | UAN, UAN+NP | PP/P, SD1 | - |  | yes | Yes |
| Drury et al. (2012) | ON/Canada | 2004-2006 | Rainfed | - | U, PCU | PP/P, SD1 | - |  | no | Yes |
| Drury et al. (2014) | ON/Canada |  | Rainfed | - | - | - | - |  | yes | Yes |
| Fujinuma et al. (2011) | MN/USA | 2009-2010 | Irrigation | - | AA, U | - | shallow, deep |  | yes | Yes |
| Gagnon et al. (2011) | MN/USA | 2004-2006 | Rainfed | 0-200 | AqA, CAN, UAN | - | - |  | yes | Yes |
| Halvorson et al. (2008) | CO/USA | 2005-2006 | Irrigation | 0-246 | - | - | - |  | yes | Yes |
| Halvorson et al. (2010a,) | CO/USA | 2007-2008 | Irrigation | - | U, PCU |  |  |  | yes | Yes |
| Halvorson et al. (2010b) | CO/USA | 2007-2008 | Irrigation | - | U, UAN, PCU, SU, UAN+AP | - | - |  | yes | Yes |
| Halvorson et al. (2011) | CO/USA | 2009-2010 | Irrigation | - | U, UAN, PCU, SU, UAN+AP | - | b’cast, band |  | yes | Yes |
| Halvorson & Del Grosso (2012) | CO/USA | 2009-2010 | Irrigation |  | U, PCU, SU |  | b’cast, band |  | yes | Yes |
| Halvorson & Del Grosso (2013) | CO/USA | 2010-2011 | Irrigation |  | U, PCU, SU |  | b’cast, band |  | yes | Yes |
| Halvorson et al. (2016a, b) | CO/USA | 2012-2014 | Irrigation | - | U, SU, DM, DM+AP | - | - |  | yes | Yes |
| Maharjan and Venterea (2013) | MN/USA | 2011-2012 | Rainfed | - | U, PCU, SU | - | b’cast, band |  | no | Yes |
| Maharjan et al. (2014) | MN/USA | 2009-2010 | Irrigation & rainfed | - | U, PCU, SU | PP/P, SD1 | - |  | no | Yes |
| Mosier et al. (2006) | CO/USA | 2002-2004 | Irrigation | 0-224 | - | - | - |  | yes | Yes |
| Omonode and Vyn (2013) | IN/USA | 2011-2012 | Rainfed | - | UAN, UAN+NP | - | - |  | yes | Yes |
| Omonode et al. (2015) | IN/USA | 2011-2012 | Rainfed | 0-220 | - | - | - |  | yes | Yes |
| Omonode et al. (2014, unpubl.) | IN/USA | 2010-2012 | Rainfed | 0-240 | UAN, UAN+NP | - | - |  | yes | No |
| Pelster et al. (2014) | QC/Canada | 2004-2005 | Rainfed | 0-160 | - | - | - |  | yes | Yes |
| Roy et al. (2014) | ON/Canada | 2011-2012 | Rainfed | 0-188 | - | PP/P, SD1 |  |  | yes | Yes |
| Sistani et al. (2011) | KY/USA | 2009-2011 | Rainfed | - |  | - | - |  | yes | Yes |
| Venterea et al. (2011) | MN/USA | 2008-2010 | Rainfed | - | U, PCU, SU, | - | - |  | no | Yes |
| Venterea and Coulter (2015) | MN/USA | 2012-2013 | Rainfed | 0-210 | - | SD1, SD2 | - |  | no | Yes |
| Zebarth et al. (2008) | NB/USA | 2004-2005 | Rainfed | 0-150 | - | PP/P, SD | - |  | no | Yes |
|  |  |  |  |  |  |  |  |  |  |  |

AA = anhydrous ammonia; AqA = aqua ammonia; AN = ammonium nitrate; PCU = polymer-coated urea; NP = nitrapyrin; SU = SUPERU®; U = urea; UAN = urea ammonium nitrate. DM = dairy manure; AP = AgrotainPlus; P = at planting; PP = pre-plant; SD1 = regular sidedress at V4-6; SD2 = late sidedress at V12-14; b’cast = broadcast.
